# Data for mitochondrial proteomic alterations in the developing rat brain

**DOI:** 10.1016/j.dib.2014.07.002

**Published:** 2014-08-12

**Authors:** Lance M. Villeneuve, Kelly L. Stauch, Howard S. Fox

**Affiliations:** Department of Pharmacology and Experimental Neuroscience, University of Nebraska Medical Center, Omaha, NE 68198, USA

**Keywords:** Mitochondria, Development, Proteomics, Oxygen tension

## Abstract

Mitochondria are a critical organelle involved in many cellular processes, and due to the nature of the brain, neuronal cells are almost completely reliant on these organelles for energy generation. Due to the fact that biomedical research tends to investigate disease state pathogenesis, one area of mitochondrial research commonly overlooked is homeostatic responses to energy demands. Therefore, to elucidate mitochondrial alterations occurring during the developmentally important phase of E18 to P7 in the brain, we quantified the proteins in the mitochondrial proteome as well as proteins interacting with the mitochondria. We identified a large number of significantly altered proteins involved in a variety of pathways including glycolysis, mitochondrial trafficking, mitophagy, and the unfolded protein response. These results are important because we identified alterations thought to be homeostatic in nature occurring within mitochondria, and these results may be used to identify any abnormal deviations in the mitochondrial proteome occurring during this period of brain development. A more comprehensive analysis of this data may be obtained from the article “Proteomic analysis of mitochondria from embryonic and postnatal rat brains reveals response to developmental changes in energy demands” in the Journal of Proteomics.

**Specifications table**

**Table:** 

Subject area	Biology
More specific subject area	Development
Type of data	Protein Expression Table
How data was acquired	SWATH Mass spectrometry; AB SCIEX Triple-TOF 5600
Data format	Normalized data
Experimental factors	Age
Experimental features	Brains were harvested from male E18 and P7 rats (*n*=4). Mitochondria were isolated by a dual, sequential isolation, and the resulting protein was used for mass spectrometry analysis
Consent	Data was obtained with approval for animal experiments provided by the University of Nebraska Medical Center IACUC
Data source location	Omaha, NE
Data accessibility	Data is provided in the paper

**Value of the data**

• These data demonstrate the ability to perform a mass spectrometry experiment with low biological variability.
• Proteins involved in many key mitochondrial processes including the electron transport chain, glycolysis, trafficking, mitophagy and the unfolded protein response were found to be differentially expressed.
• Altered mitochondrial localization can be inferred by the change in cytoplasmic proteins interacting with the mitochondria.
• Extensive proteomic alterations occur between E18 and P7 rat brain mitochondria.

## 1 Experimental design, materials and methods

Sprague-Dawley rats from Charles River were used for experiments (Wilmington, MA). Four male animals were used in the E18 and P7 groups. All protocols were conducted within NIH-approved guidelines with the approval and oversight of the University of Nebraska Medical Center IACUC.

### 1.1 Isolation of whole brain mitochondria

Brains were isolated using a previously established protocol [1] from E18 or P7 Sprague-Dawley rats (Charles River). Brains were rinsed, chopped and mitochondria were isolated as previously published using a dual sequential isolation consisting of differential centrifugation and an affinity purification with TOM22 [1,2]. Protein amount was quantified using Scopes method in conjunction with a Nanodrop (Thermo Fisher Scientific).

### 1.2 Sample preparation for mass spectrometry

Protein was treated as stated previously for mass spectrometry. In short, proteins were digesting with trypsin using FASP, and the resultant peptides were cleaned with an Oasis mixed-mode weak cation exchange cartridge (Waters, Milford, MA). A Savant ISS 110 SpeedVac Concentrator was used to concentrate samples (Thermo Fisher Scientific). A Nanodrop (Thermo Fisher Scientific) in conjunction with Scopes method was used for peptide quantification [3]. Peptides from each sample were resuspended in 6 μL of 0.1% formic acid for LC–MS/MS analysis.

### 1.3 Data-independent SWATH-MS analysis

**Fig. 1:**
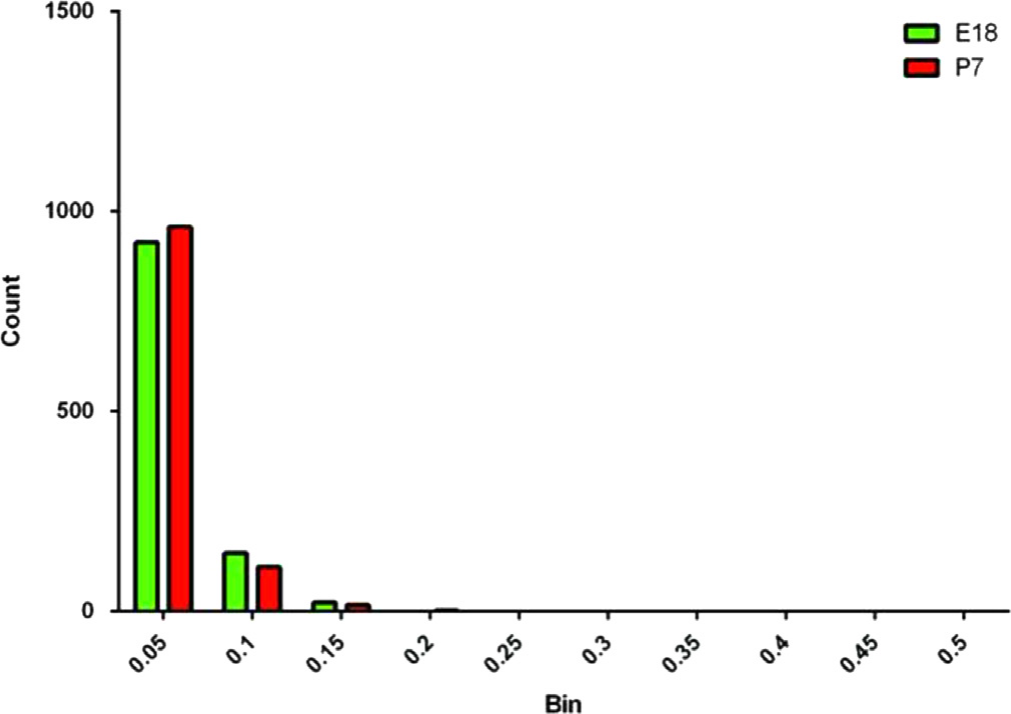
Histogram of coefficient of variation values (Log_2_ transformed). The coefficient of variation for a protein within a biological group was determined for every protein. The histogram was built of the result in Prism (Version 6.04). These results demonstrate the accuracy of SWATH-MS measurements as the coefficient of variations is low in general.

The SWATH data acquisition was performed as previously described using a nano-LC–MS/MS in conjunction with a 5600 TripleTOF instrument [1,4]. SWATH data-independent analysis (DIA) was used to analyze peptides from unfractionated E18 and P7 samples (four biological replicates per age group). Samples were run against a previously compiled SWATH library. PeakView (v. 1.1.0.0) was used to extract and integrate the ion chromatograms. Raw peak areas were generated in Peakview and exported with no data processing (neither denoising nor smoothing) of any kind applied to the extracted ion chromatograms. Retention times were calibrated using synthetic peptides (BiognoSYS; Zurich, Switzerland) that were spiked-in the samples in accordance with the manufacturer׳s protocol. In accordance with previously published work [4], 5 peptides and 5 transitions were selected for quantitative analysis and targeted data extraction for each peptide was performed. To identify peptides the fragment ion chromatograms were extracted using the SWATH isolation window set to a width of 10 min and 50 ppm accuracy for quantification purposes in accordance with previously established protocols [4]. Data was Log_2_ transformed for statistical analysis, and *t*-tests were performed between experimental groups with a Benjamini–Hochberg correction for multiple testing, using a false discovery rate of *α*<0.05 (Supplementary Table 1). Additionally, the coefficient of variation for each protein within experimental group was calculated based upon measurements in Supplementary Table 1, and a histogram made in Prism (Fig. 1; Version 6.04).

The majority of proteins were identified in both samples. Of the proteins identified, 557 of the 1094 proteins were annotated as mitochondrial according to MitoMiner (Supplementary Table 1). Protein measurements across biological replicates within the same group demonstrate small biological variability between samples (Fig. 1). The average coefficient of variation was 0.026 and 0.023 for Log_2_ transformed data for proteins from E18 and P7 animals, respectively.

A cursory analysis of the protein expression changes revealed 359 proteins had significantly altered expression in the E18 and P7 proteome (Supplementary Table 1). A large proportion of these proteins were identified as being involved in the electron transport chain, glycolysis, trafficking, mitophagy, and the unfolded protein response. In addition, there was an increase in the number of synaptic protein found in the P7 mitochondria.

## Funding sources

This work was funded by NIH MH073490 and NIH MH062261.

## Supplementary materials

Supplementary Table 1. SWATH mass spectrometry proteomic dataset of E18 and P7 rat brain mitochondria.
